# Factors Affecting Depressive Symptoms among North Korean Adolescent Refugees Residing in South Korea

**DOI:** 10.3390/ijerph14080912

**Published:** 2017-08-14

**Authors:** Subin Park, Minji Lee, Jin Yong Jeon

**Affiliations:** 1 Department of Research Planning, Mental Health Research Institute, National Center for Mental Health, Seoul 04933, Korea; subin-21@hanmail.net (S.P.); thrivingmj@gmail.com (M.L.); 2 Department of Social psychiatry and Rehabilitation, National Center for Mental Health, Seoul 04933, Korea

**Keywords:** North Korean adolescent refugees, depression, risk factor, protective factor

## Abstract

We examined factors affecting the depressive symptoms and the relationship between depression and quality of life among 131 North Korean adolescent refugees aged 12–24 years. We compared sociodemographic, social, and individual characteristics and perceived the quality of life between participants with and without depression. Thirty-seven refugees (28.2%) had clinically significant depressive symptoms. The refugees with depression were younger (*t* = 2.67; *p* = 0.009), more likely to be male (χ^2^ = 6.98; *p* = 0.009), and more likely to have a Chinese father (χ^2^ = 9.05; *p* = 0.003) than those without depression. The refugees with depression had lower levels of psychological social support (*t* = 2.96; *p* = 0.004) and resilience (*t* = 4.24; *p* < 0.001) and higher levels of alcohol problems (*t* = −2.08; *p* = 0.043), aggression (*t* = −3.15; *p* = 0.003), and post-traumatic stress disorder (PTSD; *t* = −2.89; *p* = 0.004). They also reported lower levels of life satisfaction (*t* = 3.31; *p* = 0.001) and had a more negative view of their future (*t* = 2.68; *p* = 0.010). Interventions to increase resilience, to decrease the impact of traumatic events, and to provide psychological support may be helpful for North Korean adolescent refugees at risk of depression.

## 1. Introduction

According to the Ministry of Unification of Korea [1], the number of North Korean refugees entering South Korea has rapidly increased since 1990. As of June 2017, the number is estimated to be 30,805 people, with an estimated percentage of children and youths of approximately 16%. As the number of refugees has increased, many studies have been conducted on their mental health status [2,3,4,5,6,7,8,9]. Jeon et al. [9] pointed out that many North Korean refugees have had traumatic experiences and have witnessed death by extremely dire events. They have suffered from various difficulties, not only while traveling in other countries before arriving in South Korea, but also after settling in South Korea [10,11]. As a result of these traumatic and stressful experiences, North Korean refugees are at a high risk of psychiatric symptoms [2,5,8,9,12,13].

Research on North Korean refugees tends to focus on post-traumatic stress disorder (PTSD) or depression [2,4,13,14,15], and this trend is consistent with studies on other refugees and immigrants [16,17,18,19,20]. Some studies, in addition, have found risk and protective factors that outweigh the impact of traumatic experiences on mental health [21,22,23]. For instance, researchers have sought to determine the factors that might affect depressive symptoms and their underlying mechanisms. Sociodemographic factors, such as age [5,24,25], sex [9,12], and family member-related factors [2], and social and individual factors, including social support [9,18], traumatic experiences, PTSD symptoms [14], and resilience [26,27], have been found to be related to depressive symptoms. Some researchers have found that depressive symptoms may affect the quality of life [2,4].

Because most studies conducted on North Korean refugees and the policies dealing with these refugees have mainly focused on mental health and the quality of life in adults, there is a need for research on adolescents [4,6]. Considering that adolescence is a period of distinctive physical and psychological development and further psychopathological development [28], and that refugees have had unique experiences during preflight, flight, and resettlement [29], it is not difficult to imagine that North Korean adolescent refugees face various difficulties [30]. Therefore, it is necessary to identify the risk factors and the protective factors affecting depression in North Korean adolescent refugees and to investigate how we could intervene to improve their quality of life.

On the basis of these prior studies, this study hypothesizes that sociodemographic, social, and individual characteristics affect refugees’ depressive symptoms, which are also related to their quality of life. We found that it was necessary to conduct within-group comparative studies that could guarantee the homogeneity of the group in identifying variables related to depression among North Korean adolescent refugees. Therefore, the current study was conducted to investigate factors that might affect refugees’ depressive symptoms and the impact of these on the quality of life to obtain insight for appropriate interventions and services in accordance with the results of this study.

## 2. Materials and Methods

### 2.1. Participants and Procedure

This study was conducted at three alternative schools for North Korean adolescent refugees who were preparing for the qualification examinations for middle and high school graduation in 2016. After consulting with the principals of the participating schools and the teachers in charge, the research manager visited the schools and explained the purpose of the survey to the teachers and the students. The survey was conducted on participants who voluntarily agreed to participate in the survey. All participants were asked to complete a questionnaire to obtain information on sociodemographic (age, sex, paternal origin, and residence), social (psychological and practical support), and individual (resilience, aggression, alcohol-related problems, and PTSD symptoms) characteristics; the perceived quality of life; and depression. The study was reviewed and approved by the institutional review board of the National Center for Mental Health (No. 116271-2017-22).

Among 151 students aged 12–34 years who were registered in the three participating schools, 136 students aged 12–24 years were initially selected for the study. Data from 131 participants (51 males and 80 females; mean age: 18.47 years; SD = 2.82) were used in the analysis, excluding data from 5 students who refused to participate or who answered the questions incompletely. The mean Children’s Depression Inventory (CDI) score of the participants was 16.47, with a standard deviation of 7.72. Among them, 37 (28.2%) had clinically significant depressive symptoms.

### 2.2. Measurements

#### 2.2.1 Sociodemographic Data

Sociodemographic data were gathered on age, sex, parental origin (Korean, Chinese, or other), and residence (residence with family, living with relatives/friends/alone, or residence in a facility).

#### 2.2.2. Psychological and Practical Support

Psychological support was assessed by the following question: “How much psychological support do you currently receive from your family, relatives, friends, and others around you?” Practical support was assessed by the following question: “How much practical support do you currently receive from your family, relatives, friends, and others around you?” The responses to both questions were assessed by a 10-point Likert scale (1 = “not at all”; 10 = “receive enough support”).

#### 2.2.3. Resilience

The Brief Resilience Scale [31] was used to assess resilience as the self-perceived ability to bounce back from stress. The six items were scored on a five-point scale (1 = “strongly disagree”; 5 = “strongly agree”). The scale comprised positive (items 1, 3, and 5) and negative items (items 2, 4, and 6). The range of total possible scores was from 6 to 30, and a higher score indicated a higher resilience. In our study, Cronbach’s α for the scale was 0.79.

#### 2.2.4. Aggression

Aggression was assessed using the Aggression Questionnaire (AQ) [32]. The AQ consisted of 27 items and 4 subscales: physical aggression (9 items), verbal aggression (5 items), anger (8 items), and hostility (8 items). The responses to each item were on a Likert-type scale ranging from 1 to 5 (1 = “never”; 5 = “always”). Items 15 and 24 were reverse-scoring items, and higher scores indicated a higher aggression. In our study, Cronbach’s α for the scale was 0.87.

#### 2.2.5. PTSD Symptoms

The Children’s Revised Impact of Event Scale (CRIES) [33] was used to assess PTSD symptoms. The Korean version of the CRIES was obtained from the Children and War Foundation Web page (http://www.childrenandwar.org/measures/). The scale is a self-reported questionnaire, originally adapted from the Impact of Event Scale (IES) [34]. CRIES-13 consists of 13 items assessing the core symptoms of PTSD: intrusion, avoidance, and arousal. Each item is scored on a four-point scale (0 = “not at all”; 3 = “often”). In the present sample, Cronbach’s α for the scale was 0.91.

#### 2.2.6. Alcohol-Related Problems

The Alcohol Use Disorders Identification Test (AUDIT) developed by the World Health Organization [35] is a useful tool for the early screening of individuals at risk of a drinking problem. Although AUDIT-K [36] is composed of 10 items, we measured the drinking behavior using 3 items (AUDIT-C) [37]: (1) “How often do you have a drink containing alcohol?” (2) “How many drinks containing alcohol do you have on a typical day when you are drinking?” (3) “How often do you have six or more drinks on one occasion?” Each item was scored on a five-point scale (0 = “never”; 4 = “4 + times/week”, “7–9 cups”, or “daily or almost daily”). In the present sample, Cronbach’s α for the scale was 0.83.

#### 2.2.7. Quality of Life

The quality of life was measured by the subjective well-being question from the Gallup World Poll [38], which was originally from the Cantril Self-Anchoring Striving Scale [39]. The participants were asked to describe their level of life satisfaction during the past (5 years ago), the present, and the future (5 years from now) on a scale of 0 to 10 (0 = “worst”; 10 = “best”).

#### 2.2.8. Depression

Depression was assessed using the CDI developed by Kovacs and Beck [40] to measure the degree of depression in children, as validated in a Korean version by Cho and Lee [41]. The CDI is a 27-item, self-rated, symptom-oriented scale that assesses mood during the previous 2 weeks. Each item is rated from 0 to 2; higher scores indicate a more severe degree of depression. We used a cut-off score of 22. Yang and colleagues [42] recommended cut-off scores of 22 for mild depression, 26 for moderate depression, and 29 for severe depression. In our study, Cronbach’s α for the scale was 0.75.

#### 2.2.9. Statistical Analyses

Descriptive statistics were obtained on the data for the sociodemographic characteristics, depression, the quality of life, and the psychological characteristics. Then, on the basis of the CDI cut-off point, the participants were divided into two groups (those with and without depression) to identify group differences in the measured variables. A χ^2^ test was performed on the qualitative variables, such as sex, paternal origin, and residence, and a *t*-test and an analysis of covariance (ANCOVA) were performed on each of the measured quantitative variables to reveal any significant differences between the groups. A binary logistic regression analysis with backward stepwise elimination was subsequently conducted using depression as the main outcome variable and the sociodemographic, social, and individual variables as the principal predictors. Pearson correlation analysis was performed to analyze the correlations between the measured quantitative variables. All the statistical analyses were performed using SPSS Statistics 21.0 [43]. Statistical significance was defined as an alpha of less than 0.05.

## 3. Results

Table 1 shows the sociodemographic characteristics of North Korean adolescent refugees with and without depression. All the participants’ mothers were ethnically Korean. Significant differences were found between the two groups. North Korean adolescent refugees with depression were younger (*t* = 2.67; *p* = 0.009), more likely to be male (χ^2^ = 6.98; *p* = 0.009), and more likely to have a Chinese father (χ^2^ = 9.05; *p* = 0.003) than those without depression. There was no significant difference in residence between the two groups.

Table 2 shows the social and individual characteristics of North Korean adolescent refugees with and without depression. Those with depression had lower levels of psychological social support (*t* = 2.96; *p* = 0.004) and resilience (*t* = 4.24; *p* < 0.001) and higher levels of alcohol-related problems (*t* = −2.08; *p* = 0.043), aggression (*t* = −3.15; *p* = 0.003), and PTSD symptoms (*t* = −2.89; *p* = 0.004) than those without depression. These differences remained significant even after adjustments for the age, sex, and paternal origin, except for the group difference in aggression. There was no significant difference in practical support between the groups.

Table 3 presents the perceived quality of life during the past, the present, and the future in the groups. North Korean adolescent refugees with depression reported a lower life satisfaction in the present (*t* = 3.31; *p* = 0.001) and had a more negative view of their future (*t* = 2.68; *p* = 0.010) than those without depression. The difference in life satisfaction in the past between the two groups was marginally significant (*t* = 1.90; *p* = 0.059). These differences remained significant even after adjustments for the age, sex, and paternal origin in the ANCOVA model.

Table 4 presents the results of the stepwise multiple logistic regression analysis to investigate which factors were important for predicting depression among North Korean adolescent refugees. An older age (OR = 0.78; 95% CI = 0.64–0.94), psychological support (OR = 0.75; 95% CI = 0.63–0.91), and resilience (OR = 0.83; 95% CI = 0.73–0.96) were associated with a decreased risk of depression, and alcohol-related problems (OR = 1.29; 95% CI = 1.08–1.55) were associated with an increased risk of depression.

Table 5 presents the correlations between each of the social and individual variables, the depressive symptoms, and the life satisfaction. The CDI scores negatively correlated with the psychological support (*r* = −0.21; *p* = 0.017), resilience (*r* = −0.55; *p* < 0.001), and life satisfaction (*r* = −0.45; *p* < 0.001), and positively correlated with aggression (*r* = 0.35; *p* < 0.001), alcohol-related problems (*r* = 0.21; *p* = 0.018), and PTSD symptoms (*r* = 0.43; *p* < 0.001).

## 4. Discussion

As expected, there were significant differences in the sociodemographic, social, and individual characteristics between North Korean adolescent refugees with and without depression. The higher prevalence of depression in males was inconsistent with the results of previous studies showing that the prevalence of depression in women was higher than that in men among the general population [44,45] and among North Korean refugees [12]. This discrepancy could be partially explained by sex differences in adolescent development. Pak [46] found that the mean height-for-age and weight-for age *z*-scores of North Korean refugee boys were significantly lower than those of girls, indicating that the girls’ growth status was better than that of the boys. Given the importance of growth among adolescents for comparisons with their peers, their physical growth status might influence their emotions. Another possible explanation for this discrepancy is sex differences in perceived discrimination, which is a well-known stressor of depression. Kim and Noh [47] found that men reported higher levels of discrimination, which were significantly associated with an increased risk of depressive symptoms, than women among Korean immigrants residing in Toronto, Canada. This finding implies that men are more likely to be susceptible to negative social cues, such as discrimination, and therefore, they are more vulnerable to depression.

The younger age of participants with depression was supported by the findings of previous studies. Qouta et al. [24] examined the relationships among war trauma, maternal neuroticism, psychological distress, and child psychological distress, and found that a younger age was associated with an internalizing of problems. Because their cognitive inhibitory processes are deficient and their ability to interpret the meanings of traumatic experiences and the related emotions is limited [48], younger people are more vulnerable to traumatic experiences. In addition, there was a significant difference in the paternal origin between participants with and without depression in our study; those who had a Chinese father tended to be more depressed. One explanation for the group difference in the paternal origin is that children and adolescents born to a North Korean refugee mother and a Chinese father in a third country (China) are more likely to experience social discrimination and exclusion from institutional support, a limitation in Korean language skills, family instability, and self-identity confusion [49,50,51]. Another explanation is that communication and interaction between North Korean refugee children and their Chinese fathers may be limited because the fathers and the children have different mother tongues, traditions, and values. In this case, it could be difficult for North Korean refugee children to develop a secure attachment and close relationship with their fathers, which could eventually lead to a failure of the fathers to provide appropriate guidance to their children and adolescents. In relation to this assumption, a supportive study proved the relationship between attachment and children’s depressive symptoms among North Korean refugees [25]. The refugees’ place of residence showed no significant relation with depressive symptoms, a result consistent with that of the previous study [26].

There were significant differences in individual characteristics between the depressed and non-depressed groups. The depressed group had higher levels of aggression, alcohol-related problems, and PTSD symptoms. This result was consistent with those of the previous study showing that internalized problems, including depression and anxiety, were strongly correlated with PTSD symptoms and externalized problems, including aggression and delinquency, among North Korean refugee children [51]. Our results were also consistent with previous studies showing that PTSD symptoms and depressive symptoms were related in adult North Korean refugees [14,52]. Traumatic events that North Korean adolescent refugees have experienced are possibly associated not only with PTSD symptoms but also with externalized problems, such as aggression and alcohol-related problems, and eventually influence their depressive symptoms [53]. Therefore, PTSD symptoms, alcohol-related problems, and aggression may be risk factors or early signs of depression. Lower levels of resilience in the depressed group from our study were consistent with the results of previous studies conducted on North Korean adolescent [54] and adult refugees [27] and adolescent refugees living in South Australia [23]. Generally, resilience may play a protective role against depression among refugees and may potentially ameliorate the effects of other risk factors of depression.

The level of psychological support was significantly higher in the non-depressed group. In consistency with our results, previous studies have reported that perceived social support was related to better psychological adaptation and fewer internalized symptoms among young North Korean refugees [55,56]. Baek et al. [56] classified young North Korean refugees’ friends into North and South Koreans and found that only friendships with South Korean peers were associated with fewer internalized symptoms. Friendships with South Korean peers and their psychological support might help North Korean refugees to adjust and adapt to the South Korean society. A previous study conducted on Namibian adolescent refugees also showed that good social support ameliorated the effect of prolonged exile on depression [18]. Taken together, psychological social support may not only be a protective factor but might also be a favorable prognostic factor in depression.

Additionally, we carried out further analysis to clarify the predictive factors of depression. When all the significant variables were included in the stepwise regression model, younger age, lower psychological support, lower resilience, and alcohol-related problems significantly predicted depression in the final model. Therefore, we emphasize that these four factors are the most important predictive factors of depression for North Korean adolescent refugees.

Moreover, North Korean adolescent refugees with depression reported a lower life satisfaction for the present and the future. According to previous studies, life satisfaction buffers against negative impacts of stress and psychopathological behavior among adolescents [57], and mediates the relationship between stressful life events and internalized behaviors [58]. Gilman and Huebner [59] found that youths in a high-satisfaction group reported a significantly higher adaptive function than those in a low-satisfaction group. Therefore, it is possible that life satisfaction can affect the mental health and overall social adaptation of North Korean adolescent refugees.

The study results have important practical implications for mental health service providers and policy makers. First, the results revealed that there were differences in depressive symptoms according to sex, age, and paternal origin. Understanding the differences and their underlying meanings would be helpful in providing customized services for those who are in need. Additionally, service providers would be guided to a specific and practical intervention aiming to decrease the impacts of aggression, alcohol-related problems, and PTSD symptoms while increasing individual resilience. Second, psychological support, which should be provided by the society and community to which the individual belongs, was identified as a protective factor against depression. Given the positive effects of cross-group friendships on the well-being of minorities [60], it is necessary to establish policies that increase opportunities for North Korean adolescent refugees to interact with their South Korean peers and to receive psychological support from them.

There were some limitations to this study. One limitation was its cross-sectional design, which could not identify causal relationships between depressive symptoms and other variables. A longitudinal study on North Korean adolescent refugees is recommended to overcome this limitation. Another limitation was that our participants were recruited from specific volunteering schools and were not representative of adolescent North Korean refugees. Further research needs to consider the inclusion of a wide range of samples from across the nation. Finally, the finding of depression was based on self-reported questionnaires and not on diagnostic interviews. Although the CDI used to measure the depression is a well-validated instrument, some researchers mentioned that a qualitative, participatory study needs to be conducted, particularly for a study on refugees, to achieve an overall understanding [61].

A strength of this study was that it was designed as a within-group study to ensure the homogeneity of the groups between North Korean refugee adolescents with and without depression. Additionally, this study investigated not only the risk factors for depression but also the protective factors, including resilience, which enabled us to accumulate and enhance the information and knowledge regarding North Korean adolescent refugees who were in need, to intervene in a more appropriate manner to improve their mental health and quality of life.

## 5. Conclusions

This study examined the risk factors and protective factors that affect depressive symptoms among North Korean adolescent refugees and that affect their quality of life. One-third of all the participants had clinically significant depression. Their depressive symptoms were related to the male sex; a younger age; a Chinese father; higher levels of alcohol problems, aggression, and PTSD symptoms; and lower levels of psychological support and resilience. Refugees with depression reported lower levels of life satisfaction than those who were not depressed. The results suggest that understanding the sociodemographic factors related to depressive symptoms will be of practical help for providing mental health care to North Korean adolescent refugees. We emphasize the importance of a multifaceted approach by mental health service providers and related policy makers to increase resilience, to decrease alcohol-related problems, and to provide psychological support to North Korean adolescent refugees at risk for depression.

## Figures and Tables

**Table 1:** Sociodemographic characteristics of North Korean adolescent refugees (NKAR) with and without depression.

	NKAR without Depression (*N* = 94)	NKAR with Depression (*N* = 37)		
Characteristics	*N* (%)	*N* (%)	$\chi^{2}$*/t*	*p*
Sex, male	30 (31.9)	21 (56.8)	6.89	0.009
Age, mean (SD)	18.82 (3.01)	17.59 (2.05)	2.67	0.009
Paternal origin			9.05	0.003
Korean	56 (74.7)	11 (42.3)		
Chinese	19 (25.3)	15 (57.7)		
Residence			2.45	0.293
With family	40 (43.0)	17 (47.2)		
With relatives/friends/alone	47 (50.0)	19 (52.8)		
In a facility	6 (6.5)	0		

**Table 2:** Social and individual characteristics of North Korean adolescent refugees (NKAR) with and without depression.

Characteristics	NKAR without Depression (*N* = 94)	NKAR with Depression (*N* = 37)	*t*-Test		ANCOVA	
	Mean (SD)	Mean (SD)	*t*	*p*	*F*	*p*
Social						
Psychological support	6.28 (2.77)	4.69 (2.55)	2.96	0.004	4.23	0.042
Practical support	6.57 (2.53)	5.77 (2.58)	1.60	0.113	2.71	0.103
Individual						
Resilience	19.97 (3.87)	16.75 (3.86)	4.24	<0.001	9.15	0.003
Aggression	64.20 (13.49)	73.73 (16.32)	3.15	0.003	2,32	0.131
Alcohol-related problems	1.67 (2.31)	2.86 (3.19)	2.08	0.043	5.19	0.025
Post-traumatic stress disorder symptoms	19.43 (7.96)	24.05 (8.93)	2.89	0.004	4.28	0.041

Analysis of covariance (ANCOVA) models include age, sex, and paternal origin as covariates.

**Table 3:** Perceived quality of life of North Korean adolescent refugees (NKAR) with and without depression.

Life Satisfaction	NKAR without Depression (*N* = 94)	NKAR with Depression (*N* = 37)	*t*-Test		ANCOVA	
	Mean (SD)	Mean (SD)	*t*	*p*	*F*	*p*
Present	5.36 (1.98)	4.03 (2.25)	3.31	0.001	7.24	0.008
Past	5.12 (2.38)	4.20 (2.42)	1.90	0.059	4.77	0.031
Future	8.02 (1.95)	6.74 (2.56)	2.68	0.010	5.73	0.019

Analysis of covariance (ANCOVA) models include age, sex, and paternal origin as covariates.

**Table 4:** Risk and protective factors identified in the stepwise logistic regression model.

	ß	Standard Error	Wald Test	*p*	OR (95% CI)
Dependent Variables					
Age	−0.26	0.10	6.80	0.009	0.78 (0.64–0.94)
Psychological support	−0.28	0.09	9.17	0.002	0.75 (0.63–0.91)
Resilience	−0.18	0.07	7.00	0.008	0.83 (0.73–0.95)
Alcohol-related problems	0.26	0.09	7.83	0.005	1.29 (1.08–1.55)

The ß values are the estimated unstandardized regression coefficients; OR indicates the likelihood of depression.

**Table 5:** Correlations between the measured variables.

	Depressive Symptoms	Psycho-Logical Support	Practical Support	Resilience	Aggression	Alcohol-Related Problems	PTSD Symptoms
Psychological support	−0.21 (0.017)						
Practical support	−0.17 (0.062)	0.40 (<0.001)					
Resilience	−0.55 (<0.001)	0.17 (0.053)	0.17 (0.055)				
Aggression	0.35 (<0.001)	0.003 (0.973)	0.02 (0.842)	−0.33 (<0.001)			
Alcohol- related problems	0.21 (0.018)	0.10 (0.260)	−0.04 (0.623)	−0.18 (0.047)	0.17 (0.051)		
Post-traumatic stress disorder symptoms	0.43 (<0.001)	−0.16 (0.069)	−0.18 (0.037)	−0.51 (<0.001)	0.36 (<0.001)	0.14 (0.103)	
Present life satisfaction	−0.45 (<0.001)	0.15 (0.082)	0.20 (0.025)	0.23 (0.010)	−0.07 (0.448)	−0.19 (0.033)	−0.18 (0.044)

Pearson’s *r* (*p*-value) was presented.
